# Causal relationship, shared genes between rheumatoid arthritis and pulp and periapical disease: evidence from GWAS and transcriptome data

**DOI:** 10.3389/fimmu.2024.1440753

**Published:** 2024-09-13

**Authors:** Huili Wu, Lijuan Wang, Chenjie Qiu

**Affiliations:** ^1^ Department of Endodontics, Changzhou Stomatological Hospital, Changzhou, China; ^2^ Department of General Surgery, Changzhou Hospital of Traditional Chinese Medicine, Changzhou, China

**Keywords:** rheumatoid arthritis, pulp and periapical disease, irreversible pulpitis, Mendelian randomization, hub genes

## Abstract

**Objective:**

Patients with rheumatoid arthritis (RA) have an increased risk of developing pulp and periapical disease (PAP), but the causal relationship and shared genetic factors between these conditions have not been explored. This study aimed to investigate the bidirectional causal relationship between RA and PAP and to analyze shared genes and pathogenic pathways.

**Methods:**

We utilized GWAS data from the IEU Open GWAS Project and employed five Mendelian randomization methods (MR Egger, weighted median, inverse variance weighted, simple mode, and weighted mode) to investigate the bidirectional causal relationship between RA and PAP. Transcriptome data for RA and irreversible pulpitis (IRP) were obtained from the GEO database. Hub genes were identified through differential analysis, CytoHubba, machine learning (ML), and other methods. The immune infiltration of both diseases was analyzed using the ssGSEA method. Finally, we constructed a regulatory network for miRNAs, transcription factors, chemicals, diseases, and RNA-binding proteins based on the identified hub genes.

**Results:**

RA was significantly associated with an increased risk of PAP (OR = 1.1284, 95% CI 1.0674-1.1929, p < 0.001). However, there was insufficient evidence to support the hypothesis that PAP increased the risk of RA. Integrating datasets and differential analysis identified 84 shared genes primarily involved in immune and inflammatory pathways, including the IL-17 signaling pathway, Th17 cell differentiation, and TNF signaling pathway. Using CytoHubba and three ML methods, we identified three hub genes (HLA-DRA, ITGAX, and PTPRC) that are significantly correlated and valuable for diagnosing RA and IRP. We then constructed a comprehensive regulatory network using the miRDB, miRWalk, ChipBase, hTFtarget, CTD, MalaCards, DisGeNET, and ENCORI databases.

**Conclusion:**

RA may increase the risk of PAP. The three key genes, HLA-DRA, ITGAX, and PTPRC, have significant diagnostic value for both RA and IRP.

## Introduction

Dental pulp is a type of connective tissue located inside teeth, encased by dentin and enclosed within the pulp cavity. It communicates with periapical tissue through the apical foramen or accessory root canals. Endodontic disease, encompassing pulp and periapical diseases (PAP), was influenced by various factors such as microbial infection, stimulation from drugs and filling materials, temperature changes, electrical currents, pressure variations, and trauma (1). The initial response to pulp stimulation was inflammation. Pathogen invasion of the pulp triggered immune and inflammatory responses, leading to pulpitis. Mild invasive factors can cause reversible pulpitis, where the inflammation was controlled and the pulp was repaired. However, severe invasive factors resulted in irreversible pulpitis (IRP), ultimately causing pulp necrosis. When bacteria invaded the periapical tissue, it can lead to acute or chronic inflammation of this area (2). Endodontic disease was prevalent, with 52% of adults worldwide experiencing apical periodontitis (AP) in at least one tooth (3). Moreover, it was associated with systemic conditions such as metabolic disorders, autoimmune and cardiovascular diseases, adverse pregnancy outcomes, and mental illnesses (4, 5).

Rheumatoid arthritis (RA) is a chronic autoimmune disease with a complex pathogenesis, primarily characterized by progressive joint damage and extra-articular manifestations, leading to joint pain, stiffness, and functional impairment (6). Beyond its impact on joints, numerous recent studies have identified a potential correlation or causal relationship between RA and various oral diseases, including changes in oral microbiota (7, 8), oral health issues (9), periodontitis (10, 11), and Sjogren’s syndrome (12, 13). Notably, periodontitis and pulpitis shared similar pathogenic factors such as bacterial infection, inflammation, and immune responses (14). Early research revealed that free plasma cells producing rheumatoid factors were detected in the periapical lesions of 6% of RA patients (15). A systematic review comprising five studies found a significant correlation between RA and AP, with the incidence of AP in the RA group ranging from 1.53% to 75% (16). Another cross-sectional study reported that the incidence of AP in the RA group (4.3%) was significantly higher than in the control group (2%), with an odds ratio (OR) of 2.193 (17). Shiori et al. used SKG mice to investigate the effect of RA on immune response disruption in pulpitis and periapical periodontitis. They found that 14- and 28-days post-surgery, the number of apoptotic cells in the pulp and periapical tissues of SKG mice was higher than in the control group. This suggested that RA-related immune response disruption was associated with prolonged inflammation in pulpitis and periapical periodontitis (18). Therefore, there appears to be a potential correlation between RA and PAP, warranting further exploration into their interrelationship.

Mendelian randomization (MR) is a powerful analytical technique in epidemiological research that leverages genetic variations strongly correlated with exposure factors as instrumental variables to assess causal relationships between these factors and health outcomes. The essence of MR lies in using genetic data as a bridge to explore causality between a specific exposure and a particular outcome. Traditional observational studies often face limitations, such as the inability to fully account for reverse causality and confounding factors, which can result in biased associations and conclusions (19). Confounding factors in observational studies may create false associations between exposures and outcomes. MR mitigates this issue by using genetic variations that are randomly assigned at conception, making them unlikely to be linked with confounding factors (20). Additionally, traditional observational studies frequently struggle with reverse causality, where it remains unclear whether the exposure leads to disease or the disease affects the exposure. MR circumvents this issue because genetic variations are determined at conception and are not influenced by future disease onset (21). With the advent of large-scale GWAS, MR analysis has become more powerful and feasible, enabling researchers to explore causal relationships between various diseases and risk factors with greater precision (22). Furthermore, due to its strong evidential support and cost-effectiveness, MR provides a robust scientific foundation for understanding the relationship between genes and diseases. This, in turn, aids in the development of personalized treatment strategies and advances the field of precision medicine. Despite some cross-sectional and cohort studies examining the relationship between RA and PAP, the existing evidence remains inadequate. To address this gap, we propose to explore the causal relationship between RA and PAP using MR analysis. Additionally, advancements in high-throughput sequencing technology have led to an increase in the use of transcriptome data to analyze potential relationships and pathogenic pathways between diseases. Previous research has examined the correlation between RA and periodontitis (23). Building on this foundation, we plan to further investigate the common pathways, hub genes, and regulatory networks between RA and IRP through public databases.

## Materials and methods

### Data source and processing

**Figure 1:**
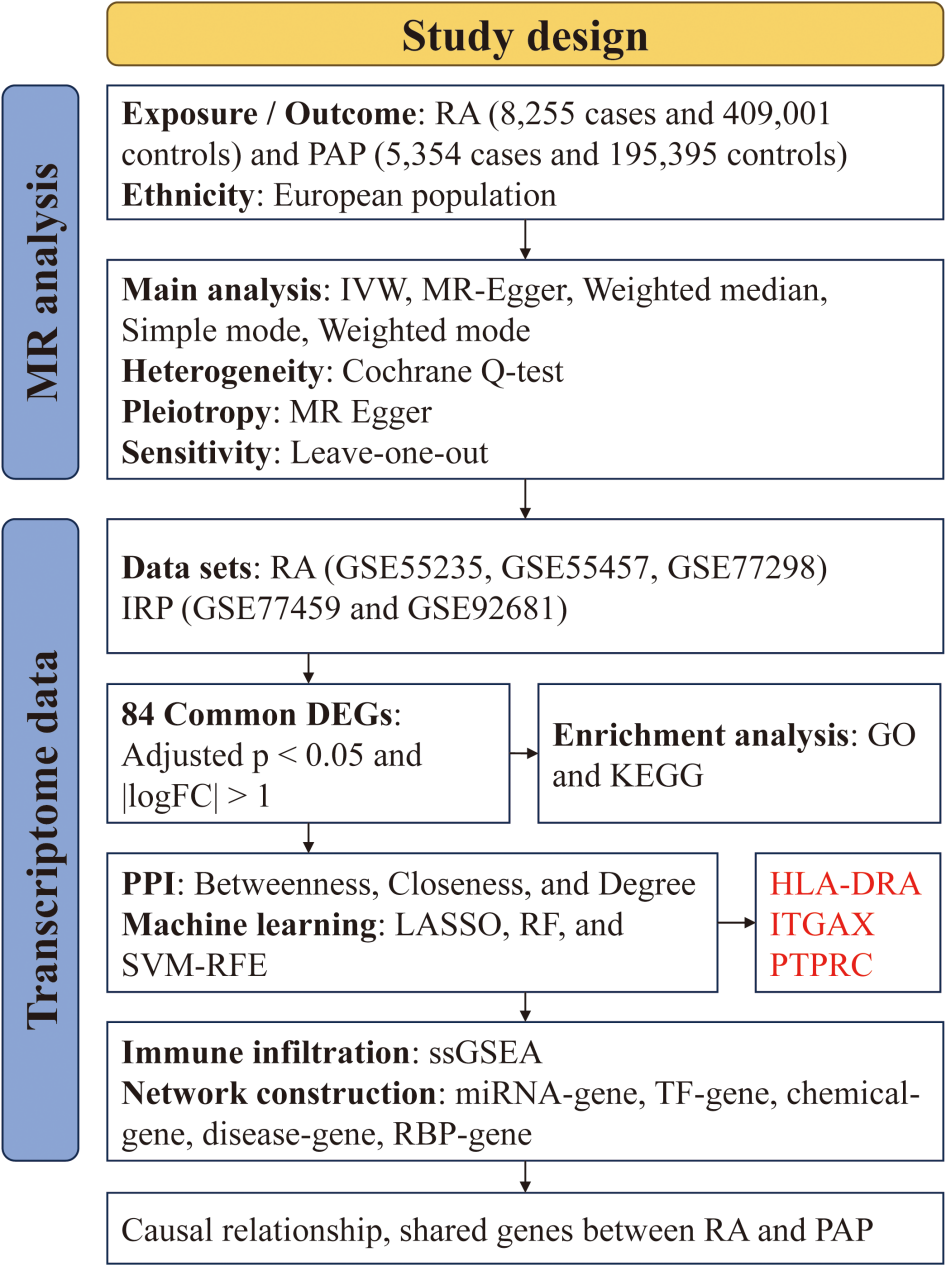
The flowchart of the study.

We acquired GWAS summary data pertaining to RA and PAP through the IEU Open GWAS Project (https://gwas.mrcieu.ac.uk/). The RA data (ID: ebi-a-GCST90018910) was retrieved from the EBI database, encompassing 8,255 cases and 409,001 controls (24). The PAP data (ID: finn-b-K11-PULP_PERIAPICAL) comprised 5,354 cases and 195,395 controls. Both datasets primarily represented European populations. Additionally, transcriptome data for RA and IRP were sourced from the GEO database (https://www.ncbi.nlm.nih.gov/geo/), alongside relevant clinical information. For RA, synovial tissue expression profiles were gathered from the GSE55235, GSE55457, and GSE77298 datasets, while for IRP, inflamed or normal pulp expression profiles were acquired from the GSE77459 and GSE92681 datasets. Subsequently, utilizing the respective platform information, gene symbols were standardized. The expression matrices underwent normalization using the “normalizeBetweenArrays” function within the “limma” package, with batch effects mitigated through the “combat” function of the “sva” package. Consequently, an integrated expression matrix for RA was established, comprising 37 negative control (NC) and 39 RA samples, while a corresponding matrix for PAP encompassed 11 NC and 13 IRP samples. The detailed information of the above dataset is shown in **Table 1**, and we have also drawn a flowchart to show the entire research process more clearly (**Figure 1**).

**Table 1:** The detailed information of included datasets in the study.

Datasets	Platform	Diseases	Cases: Controls
ebi-a-GCST90018910	–	RA	8255: 409001
finn-b-K11-PULP_PERIAPICAL	–	PAP	5354: 195395
GSE55235	GPL96	RA	10: 10
GSE55457	GPL96	RA	10: 13
GSE77298	GPL570	RA	11: 16
GSE77459	GPL17692	IRP	6: 6
GSE92681	GPL16956	IRP	5: 7

### Mendelian randomization

Based on the fundamental assumptions of MR analysis (association, independence, and exclusivity), we systematically screened Instrumental variables (IVs). Specifically, single nucleotide polymorphisms (SNPs) associated with RA were utilized for forward MR analysis, while those linked to PAP were employed for reverse MR analysis. To ensure the selection of SNPs closely linked to the exposure factors, a stringent significance threshold of p < 5 × 10^-6^ was set. Additionally, a linkage imbalance window of 10,000 kb with an r^2^ < 0.001 was applied to guarantee the independence of the chosen genetic variants. Ultimately, 59 SNPs were identified as IVs for RA, and 14 SNPs were designated as IVs for PAP (**Supplementary Tables S1**, **S2**). The robustness of the association between IVs and exposure was assessed using the F-value, with IVs possessing an F-value below 10 deemed weak and consequently excluded. Notably, all IVs selected through the aforementioned screening exhibited F-values exceeding 10 (**Supplementary Tables S1**, **S2**). Subsequently, we employed five MR methods to scrutinize the causal relationship between RA and PAP, with the IVW method serving as the primary analytical approach. Concurrently, supplementary methods including the simple model, weighted model, weighted median, and MR Egger were utilized to corroborate the robustness of the findings. Moreover, comprehensive analyses encompassing heterogeneity, pleiotropy, and sensitivity were conducted. Heterogeneity among IVs was evaluated using the Cochrane Q-test, where a significance level of p value < 0.05 indicated the presence of heterogeneity. To assess horizontal pleiotropy, MR Egger analysis, including an intercept test, was employed. A p value > 0.05 signified the absence of pleiotropy. Causal relationships were visually depicted through forest and scatter plots, while sensitivity analysis, employing the leave-one-out method to gauge the impact of individual SNPs on the results, was performed. Additionally, heterogeneity was visualized using funnel plots to provide a comprehensive assessment of the MR analysis.

### Definition of PAP, IRP and RA

The MR study utilized GWAS data from the FinnGen research project, which integrates health and genetic data from the Finnish Health Registries. The analysis was adjusted for covariates such as gender, age, genotyping batch, and the first ten genetic principal components. The PAP in this study is defined using International Classification of Diseases (ICD) codes: Hospital discharge with ICD-8: 522; ICD-9: 522; and ICD-10: K04 (14). Inflamed pulp tissues were extracted from teeth diagnosed with IRP in accordance with the endodontic diagnostic system of the American Association of Endodontists. Teeth affected by periodontitis were excluded. Patients with compromised immune systems or those on medications known to influence immune response were also excluded from the study (25, 26). In addition, RA patients were classified based on the American College of Rheumatology criteria valid during the sample assessment period (27, 28).

### Differential expression analysis

Differential expression analyses were performed between NC and RA samples, as well as between NC and IRP samples, utilizing the “limma” package. This analysis aimed to discern genes exhibiting significant differential expression, employing stringent criteria of adjusted p value < 0.05 and |log2FC| > 1. Visualization of these differentially-expressed genes (DEGs) was accomplished through volcano plots and heatmaps, aiding in the elucidation of distinct expression patterns between the studied groups.

### Enrichment analysis

The “clusterprofiler” package was employed to undertake a comprehensive functional enrichment analysis of DEGs, incorporating Gene Ontology (GO), Kyoto Encyclopedia of Genes and Genomes (KEGG), and Disease Ontology (DO) analyses. Within the GO analysis, DEGs were categorized into three distinct domains: biological processes (BP), cellular components (CC), and molecular functions (MF), thereby providing a holistic view of their functional implications. KEGG analysis was leveraged to elucidate potential pathways associated with the DEGs, offering insights into the biological mechanisms underlying the observed expression changes. Furthermore, DO enrichment analysis was instrumental in identifying diseases closely linked to the DEGs, enhancing our understanding of their pathological relevance. To ensure robust results, stringent screening criteria were applied, with significance thresholds set at a p value < 0.05 and a q value < 0.05 for the enrichment analyses.

### Protein-protein interaction network

The DEGs were utilized as input for constructing a PPI network via the STRING website (https://cn.string-db.org/), with a minimum required interaction score set at 0.40. Subsequently, these interactions were imported into Cytoscape software to identify potential candidate genes. Initially, the MCODE plug-in was employed to identify significant modules within the network, utilizing a cluster score threshold greater than 3. Functional annotation via KEGG analysis was performed on these modules to elucidate potential biological functions. Next, three centrality algorithms (Betweenness, Closeness, and Degree) within the CytoHubba plug-in were utilized to score the nodes in the PPI network, aiming to identify the top 20 genes based on their importance in network connectivity. The union of genes obtained through these algorithms constituted the pool of candidate important genes for further analysis.

### Machine learning algorithms

Three ML algorithms, namely the least absolute shrinkage and selection operator (LASSO), support vector machine - recursive feature elimination (SVM-RFE), and random forest (RF), were harnessed to uncover hub genes associated with IRP. LASSO regression, grounded in the principles of linear regression, leverages gene expression profiles to pinpoint pivotal genes. Dimensionality reduction was achieved through the “glmnet” package, employing the smallest lambda value as the threshold. SVM-RFE ranks features based on their scores obtained from training the model, iteratively eliminating features with the lowest scores until the desired number of features is reached, aligning with the maximum margin principle of SVM. In our investigation, SVM-RFE was implemented using the “e1071” package. RF, recognized for its adeptness in sifting through extensive datasets to identify the gene set with minimal error rate, was employed to accurately identify potential disease biomarkers. Candidate genes were scrutinized using the “randomForest” package, and the top 10 genes with relative importance scores were designated as key features. Subsequently, an intersection operation was conducted on the selected genes identified by the three algorithms to procure stable and comprehensive diagnostic markers for IRP (29).

### Immune infiltration evaluation

Leveraging the expression profiles, we examined the relative infiltration levels of 23 immune cell types employing the ssGSEA algorithm, implemented via the “GSVA” package. Disparities in immune infiltration between NC and disease samples were scrutinized through heatmaps and violin plots. In order to delve into the influence of diagnostic markers on immune infiltration regulation in IRP and RA, correlation analysis was conducted between diagnostic markers and immune cells.

### Regulatory network construction

To delve deeper into the potential roles of three hub diagnostic genes, we constructed an extensive array of regulatory networks, encompassing miRNA-gene, transcription factor (TF)-gene, chemical-gene, disease-gene, and RNA binding protein (RBP)-gene networks. Initially, we employed the miRDB and miRWalk databases, imposing a screening condition of a minimum of 20 pairings, to identify miRNAs regulating the hub genes. MiRNAs intersecting between the two databases were deemed to possess regulatory potential over the hub genes. Subsequently, we utilized the hTFtarget and ChipBase databases to identify common TFs and construct a regulatory network for TF-mRNA interactions. For predicting potential drugs targeting the hub genes, we leveraged the CTD database with an interaction count ≥ 2. Additionally, to pinpoint diseases closely associated with the hub genes, we utilized the DisGeNET and MalaCards databases to identify common diseases and construct disease networks. Furthermore, to predict the potential binding of RBPs to hub mRNAs, we explored the ENCORI database, setting the screening criteria to CLIP data from ≥ 1 dataset, thereby identifying RBPs with binding potential to hub genes. The aforementioned five regulatory relationships were integrated into Cytoscape software for network visualization and construction.

### Statistical analysis

The Wilcoxon test was utilized to compare continuous variables between the two groups, assessing differences in a robust and non-parametric manner. To gauge the diagnostic efficacy of markers, the area under the curve (AUC) of the receiver operating characteristic (ROC) was computed. Spearman’s test was employed to scrutinize correlations between continuous variables, offering insights into potential associations. All analyses and visualizations were performed either using R software or via an online platform, ensuring comprehensive and accessible data exploration. A significance threshold of p value < 0.05 was applied for statistical inference.

## Results

### Mendelian randomization analysis

**Figure 2:**
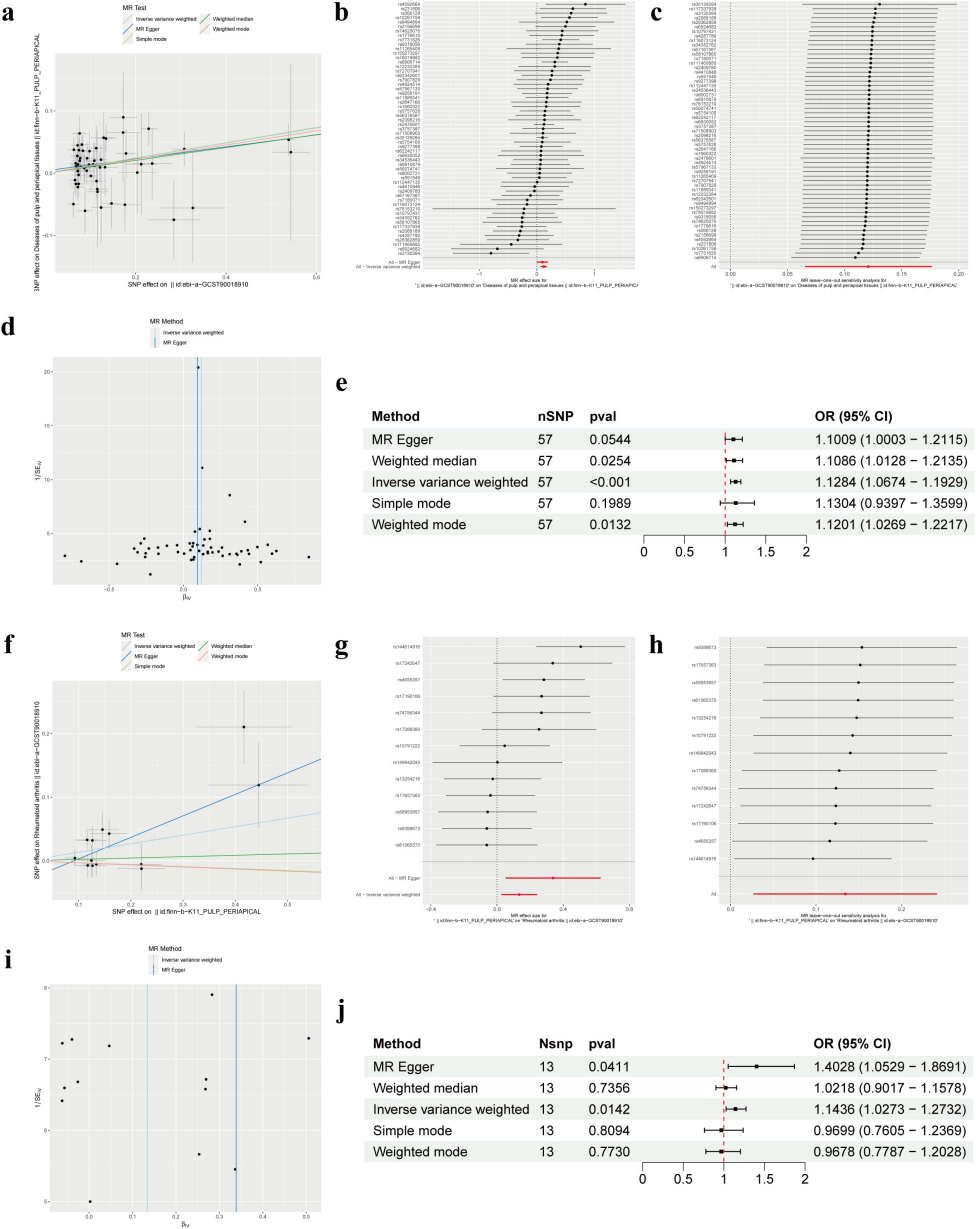
Mendelian randomization revealed the bidirectional causal relationship between RA and PAP. **(A)** Scatter plot of SNPs related to RA and the risk of PAP. **(B)** Forest plot of SNPs associated with RA and the risk of PAP. **(C)** Leave‐one‐out of SNPs associated with RA and the risk of PAP. **(D)** Funnel plot for RA on PAP. **(E)** Associations of genetically predicted RA and the risk of PAP. **(F)** Scatter plot of SNPs related to PAP and the risk of RA. **(G)** Forest plot of SNPs associated with PAP and the risk of RA. **(H)** Leave‐one‐out of SNPs associated with PAP and the risk of RA. **(I)** Funnel plot for PAP on RA. **(J)** Associations of genetically predicted PAP and the risk of RA. RA, Rheumatoid arthritis; PAP, Pulp and periapical disease; SNP, Single nucleotide polymorphism.

Our forward MR analysis delving into the causal relationship between RA and PAP yielded noteworthy findings. We uncovered a significant correlation, demonstrating that RA was associated with an increased risk of PAP (**Figures 2A, B**). Employing the IVW method, our investigation revealed a causal link between RA and PAP (OR = 1.1284, 95% CI 1.0674-1.1929, p < 0.001). This outcome was corroborated by both the Weighted Median method (OR = 1.1086, 95% CI 1.0128-1.2135, p = 0.0254) and the Weighted Mode method (OR = 1.1201, 95% CI 1.0269-1.2217, p = 0.0132) (**Figure 2E**). Notably, Cochrane’s Q-test indicated no significant heterogeneity among the included IVs (p > 0.05). Further support for our findings emerged from MR Egger regression (intercept t = 0.0039, p = 0.536), which confirmed the absence of horizontal pleiotropy (**Table 2**). Additionally, sensitivity analysis demonstrated the robustness of our results, with no significant alterations upon exclusion of any single SNP, thus enhancing the credibility and stability of our MR findings (**Figure 2C**). Furthermore, the funnel plot exhibited symmetrical distribution around both sides of the IVW and MR Egger analyses (**Figure 2D**).

**Table 2:** Results of sensitivity analyses.

Exposure	Outcome	Heterogeneity (MR Egger)		Heterogeneity (IVW)		Horizontal pleiotropy		
		Q	Q_pval	Q	Q_pval	Egger intercept	se	pval
RA	PAP	58.45	0.3498	58.87	0.3711	0.0039	0.0062	0.536
PAP	RA	17.25	0.1006	20.75	0.05412	-0.031	0.021	0.163

In our MR analysis exploring the causal relationship between PAP and RA, we did not identify a significant correlation between PAP and RA (**Figures 2F, G**). Utilizing the IVW method, we discerned a causal association between PAP and RA (OR = 1.1436, 95% CI 1.0273-1.2732, p = 0.0142), which was supported by the MR Egger method (OR = 1.4028, 95% CI 1.0529-1.8691, p = 0.0411) (**Figure 2J**). However, the effect values of simple mode and weighted mode went in the opposite direction. Cochrane’s Q-test revealed no significant heterogeneity among the incorporated IVs (p > 0.05). Additionally, MR Egger regression (intercept t = -0.031 p = 0.163) lent further credence to our findings by indicating the absence of horizontal pleiotropy (**Table 2**). Consistent with our earlier analysis, sensitivity testing affirmed the reliability of our results, with no notable changes upon exclusion of any single SNP (**Figure 2H**). Moreover, the funnel plot exhibited symmetrical distribution around both sides of the IVW analysis (**Figure 2I**).

### Differential expression analysis

**Figure 3:**
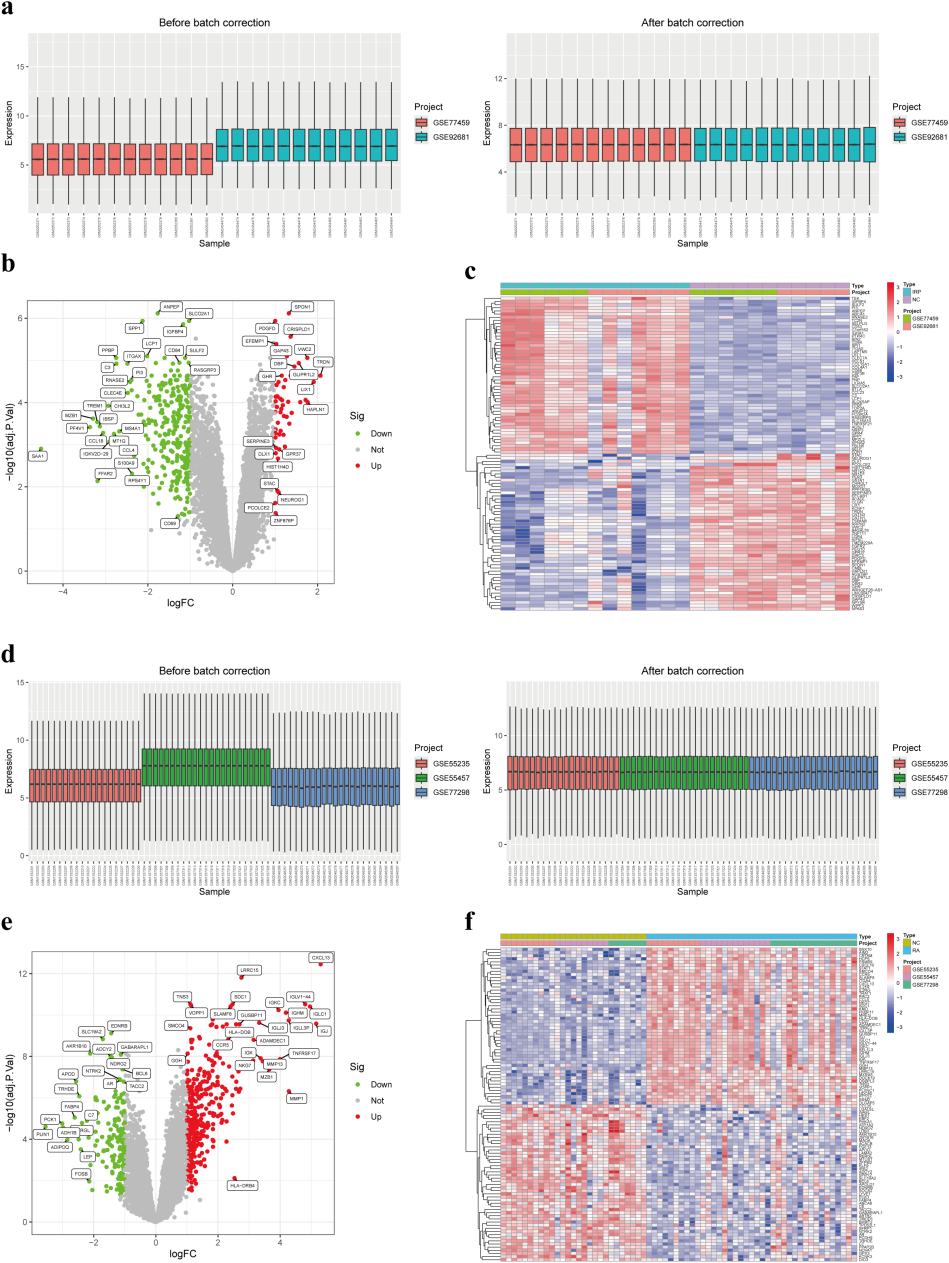
Identification of DEGs in IRP and RA. **(A)** Box plots of two IRP datasets before and after batch correction. **(B)** Volcano plot of the DEGs in IRP. **(C)** Heatmap of the top 50 up-regulated and down-regulated genes in IRP. **(D)** Box plots of three RA datasets before and after batch correction. **(E)** Volcano plot of the DEGs in RA. **(F)** Heatmap of the top 50 up-regulated and down-regulated genes in RA. DEGs, Differentially-expressed genes; IRP, Irreversible pulpitis.

Utilizing two distinct datasets, GSE77459 and GSE92681, a cohort comprising 24 samples including both NC and IRP samples underwent a rigorous normalization process coupled with batch correction. Visual representation through box plots vividly illustrated the discernible disparities pre- and post-batch correction, affirming the successful mitigation of batch effects (**Figure 3A**). Employing the “limma” package for differential analysis, we identified a collective of 332 DEGs, with 281 exhibiting upregulation in IRP samples and 51 displaying downregulation (**Figures 3B, C**). Similarly, our investigation encompassed three datasets, namely GSE55235, GSE55457, and GSE77298, incorporating a combined total of 66 samples inclusive of both NC and RA samples, which underwent analogous batch correction procedures. Visual scrutiny via box plots unveiled the successful alleviation of batch effects subsequent to correction (**Figure 3D**). Subsequent differential analysis, supplemented by volcano and heatmap, facilitated the identification of 505 genes exhibiting aberrant expression in RA samples. Of these, 344 genes were upregulated while 161 genes were downregulated in RA (**Figures 3E, F**). Consequently, we plan to pursue a comparative analysis by intersecting the DEGs identified in both diseases to glean further insights.

### Enrichment analysis

**Figure 4:**
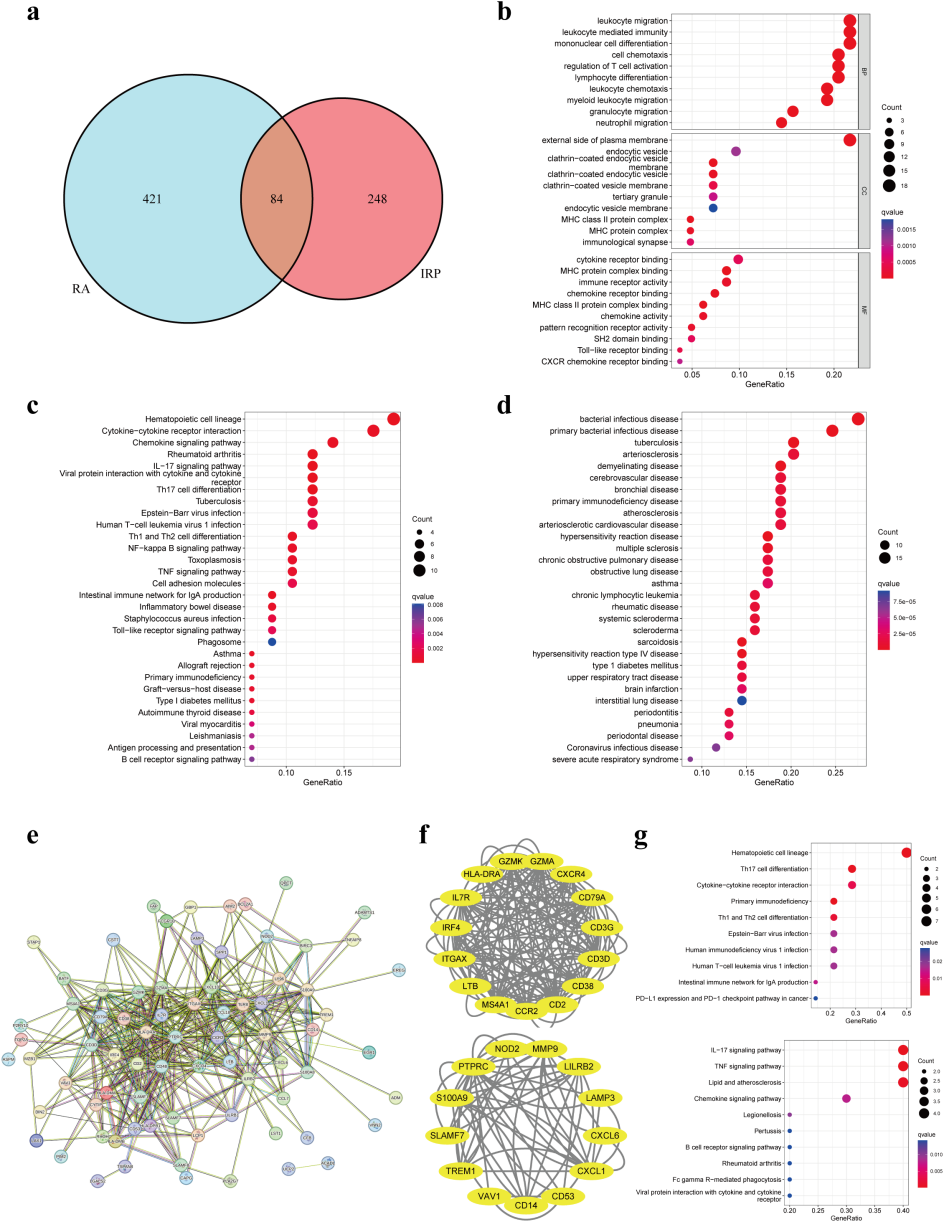
Enrichment analyses based on the shared DEGs. **(A)** Intersecting DEGs obtained by IRP group and RA group. **(B)** GO analysis according to the DEGs. **(C)** KEGG analysis revealed the pathways related to the DEGs. **(D)** DO analysis revealed the diseases related to the DEGs. **(E)** Construction of a PPI network using the proteins encoded by the 84 DEGs. **(F)** Identification of two important modules using the MCODE plug-in. **(G)** KEGG analyses for the two modules. GO, Gene Ontology; KEGG, Kyoto Encyclopedia of Genes and Genomes; DO, Disease Ontology; PPI, Protein-protein interaction.

Through the intersection of DEGs, a comprehensive set of 84 genes was identified, showcasing aberrant expression patterns in both diseases (**Figure 4A**). Functional enrichment analysis elucidated their involvement primarily in processes pertinent to leukocytes or lymphocytes, including but not limited to leukocyte migration, leukocyte-mediated immunity, and mononuclear cell differentiation. Furthermore, CC analysis unveiled associations with endocytic vesicles or MHC protein complexes. Notably, MF analysis underscored significant enrichment of DEGs in immune-related binding activities (**Figure 4B**). KEGG pathway analysis offered insights into the intricate involvement of these DEGs in immune-related pathways such as cytokine signaling, chemokine signaling, IL-17 signaling, as well as differentiation pathways of T-helper cell subsets (Th1, Th2, and Th17). Additionally, these pathways were linked to immune-related disorders including rheumatoid arthritis, inflammatory bowel disease, asthma, type 1 diabetes, and autoimmune thyroid disease (**Figure 4C**). Furthermore, DO analysis corroborated the association of DEGs not only with bacterial infectious diseases but also with a spectrum of immune-related disorders (**Figure 4D**). Collectively, these findings suggest a plausible correlation between the co-occurrence of the two diseases and the infiltration of immune cells such as leukocytes, as well as the dysregulation of immune-related pathways.

### Hub genes identification

We employed the identified DEGs to construct a PPI network via the STRING website. The resulting network exhibited a substantial complexity, comprising 84 nodes interconnected by 399 edges, indicative of extensive protein interactions (**Figure 4E**). Subsequent analysis utilizing the MCODE plug-in allowed us to discern two significant modules within the network. Module 1 prominently featured associations with hematopoietic cell lineage, Th17 cell differentiation, cytokine-cytokine receptor interaction, and primary immunodeficiency pathways. Conversely, Module 2 showcased connections with IL-17, TNF, and chemokine signaling pathways, alongside pathways related to lipid metabolism and atherosclerosis (**Figures 4F, G**).

**Figure 5:**
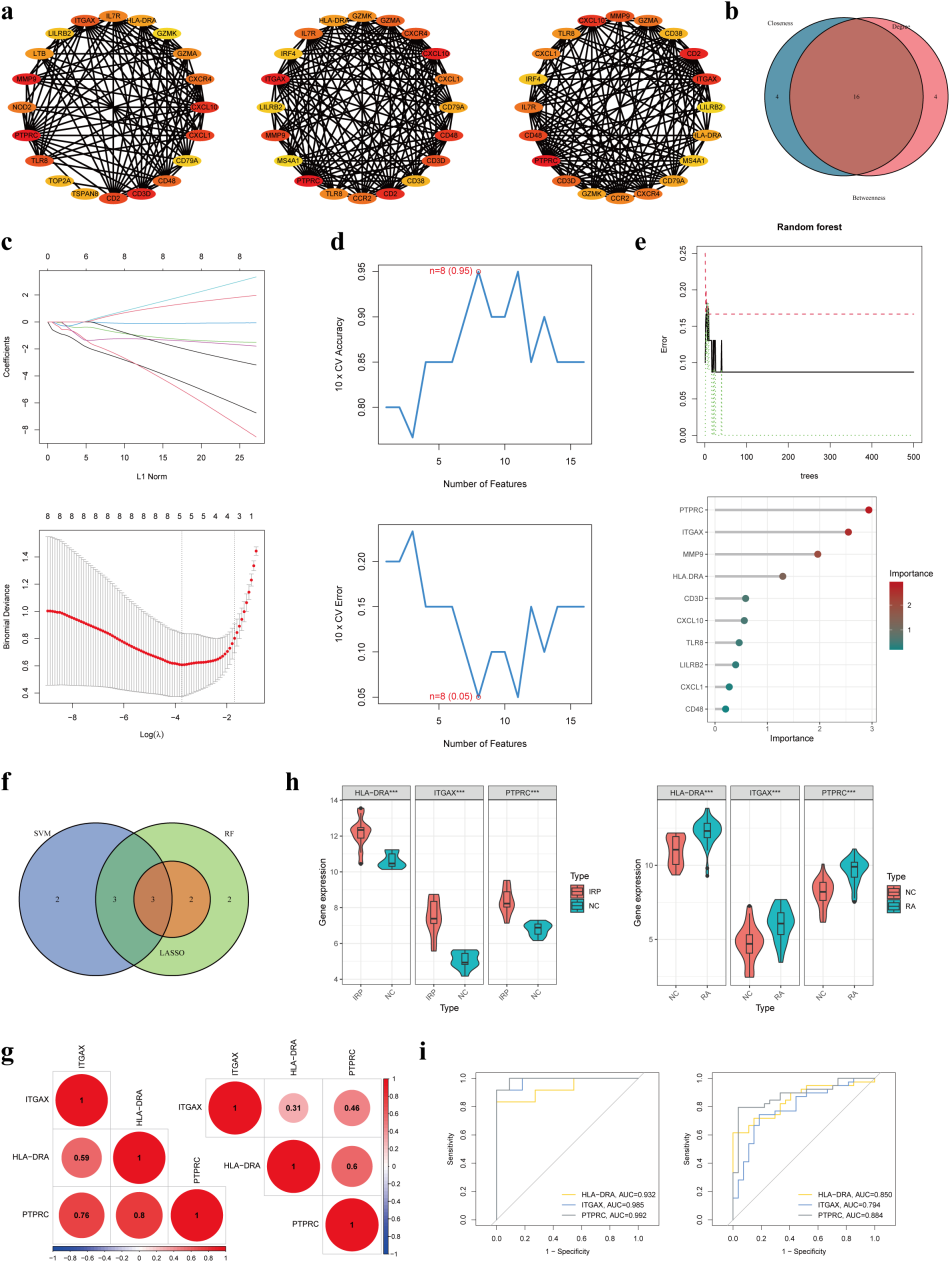
Identification of three hub genes in IRP. **(A)** Three algorithms (Betweenness, Closeness, and Degree) within the CytoHubba plug-in presented the top 20 genes in the PPI network. **(B)** Intersecting the key genes obtained by the above three algorithms. **(C)** Identification the key genes among the 16 genes using LASSO regression. **(D)** Identification the key genes using SVM-RFE. **(E)** Identification the key genes using RF. **(F)** Obtaining the three hub genes using the three ML methods. **(G)** The correlation among the three genes in IRP and RA, respectively. **(H)** Verification of the differential expression of the hub genes in IRP and RA groups. **(I)** Verification of the diagnostic value of the hub genes using the ROC curves. LASSO, least absolute shrinkage and selection operator; SVM-RFE, Support vector machine-recursive feature elimination; RF, Random forest; ML, Machine learning; ROC, Receiver operating characteristic. P***<0.001.

Following this, we applied three distinct topological network algorithms sourced from the CytoHubba plug-in to generate a subnetwork composed of the top 20 genes identified by each algorithm (**Figure 5A**). Through the amalgamation of these outcomes, a total of 16 genes emerged, appearing consistently in the output of all three algorithms (**Figure 5B**). Subsequently, these 16 genes underwent hub gene identification employing three diverse ML algorithms. Utilizing LASSO regression, we pinpointed 5 core genes (**Figure 5C**), while SVM-RFE revealed 8 core genes with an impressive accuracy rate of 0.95 and an error rate of 0.05 (**Figure 5D**). Additionally, employing the RF method, the top 10 genes based on importance were designated as core genes (**Figure 5E**). Upon scrutinizing the outcomes, we observed an intersection highlighting three hub genes (HLA-DRA, ITGAX, and PTPRC) in the context of IRP (**Figure 5F**). Subsequent correlation analysis of these three genes in both IRP and RA samples unveiled significant associations, particularly notable between HLA-DRA and PTPRC (**Figure 5G**). A comprehensive evaluation of the diagnostic potential of these three hub genes across IRP and RA samples was conducted. Visual examination revealed markedly elevated expression levels of the three genes in both IRP and RA groups, with AUC values exceeding 0.9 in IRP and surpassing 0.8 in RA (**Figures 5H, I**).

### Immune infiltration

**Figure 6:**
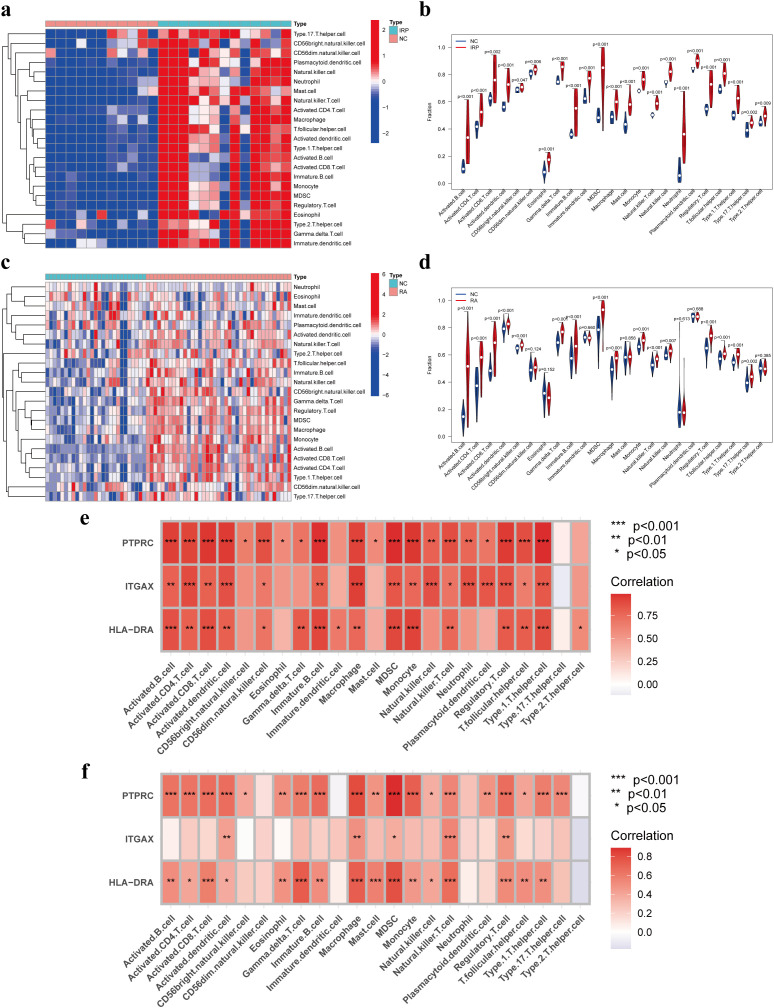
Immune infiltration analysis of IRP and RA groups. Comparison of the fraction of 23 immune cells between NC and IRP samples using the heatmap **(A)**, and the violin plot **(B)**. Comparison of the fraction of 23 immune cells between NC and RA samples using the heatmap **(C)**, and the violin plot **(D)**. The relationship between the hub genes and immune cell infiltration in IRP **(E)** and RA **(F)**, respectively. NC, Negative control. P*<0.05, P**<0.01; P***<0.001.

Prior studies have elucidated the pivotal roles of HLA-DRA, ITGAX, and PTPRC in orchestrating immune cell infiltration. Building upon this foundation, our investigation aimed to elucidate the intricate interplay between these genes and immune cells. Initially, we conducted a comparative analysis of the infiltration levels of 23 distinct immune cell types between NC and IRP samples, as well as between NC and RA samples, employing ssGSEA (**Figures 6A, C**). Notably, the violin plots unveiled significantly augmented levels of nearly all immune cell types in both IRP and RA samples relative to NC samples (**Figures 6B, D**). Subsequent correlation analysis unveiled positive associations between HLA-DRA, ITGAX, PTPRC, and various immune cell subsets including activated dendritic cells, macrophages, MDSCs, natural killer T cells, and regulatory T cells, observed consistently in both IRP and RA samples (**Figures 6E, F**).

### Regulatory network

**Figure 7:**
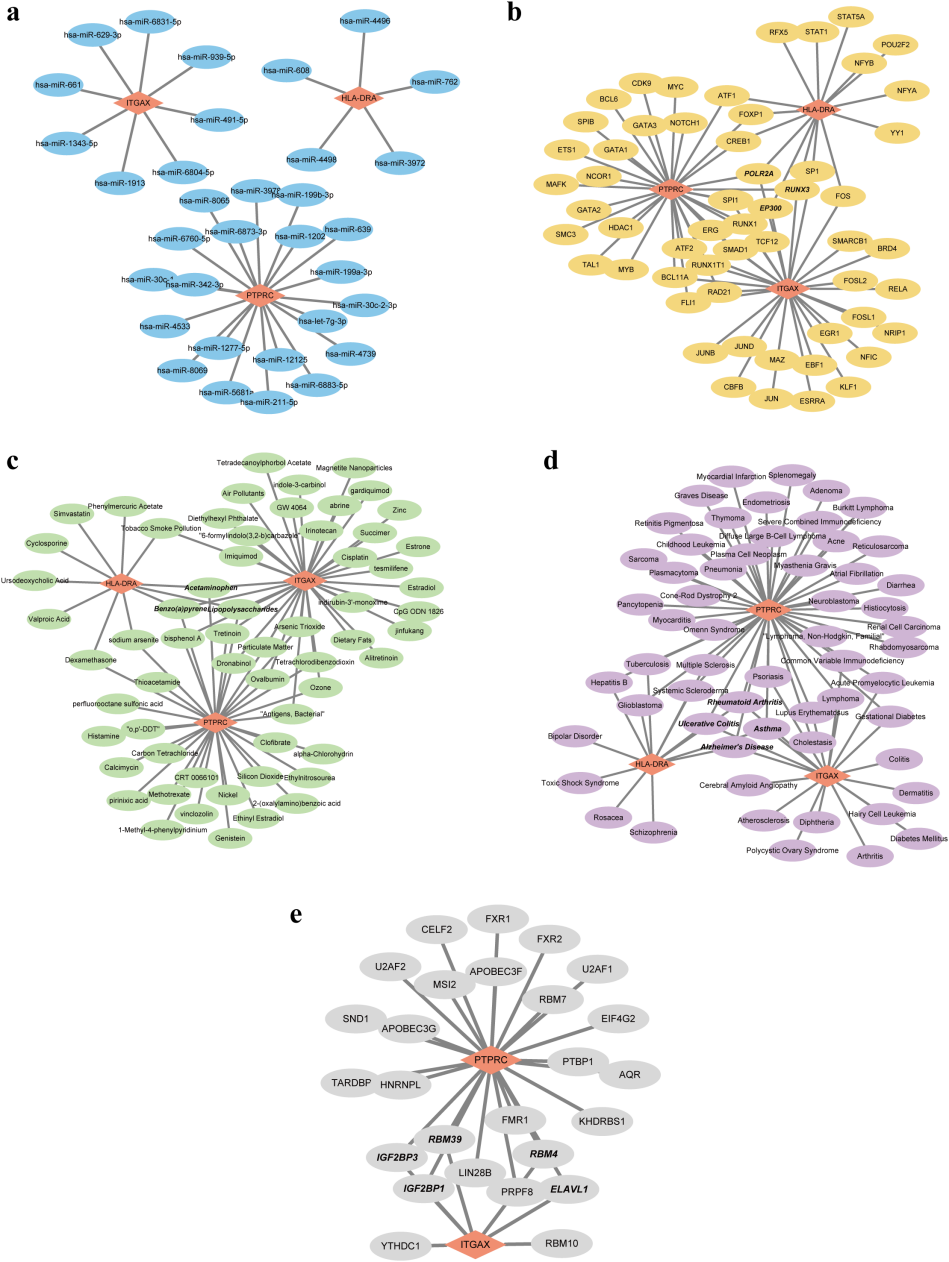
Five regulatory network of the three hub genes. **(A)** Visualization of the miRNA-mRNA network. **(B)** Visualization of the TF-mRNA network. **(C)** Visualization of the chemical-mRNA network. **(D)** Visualization of the disease-mRNA network. **(E)** Visualization of the RBP-mRNA network. TF, Transcription factor; RBP, RNA-binding protein.

Based on the pivotal roles of three hub genes, we embarked on predicting potential regulatory elements including miRNAs, TFs, chemicals, diseases, and RBPs associations to delineate the multifaceted regulatory landscape. Initially, leveraging the miRDB and miRWalk databases, we forecasted a total of 33 miRNAs with no overlapping candidates. Subsequently, we constructed a comprehensive miRNA-mRNA regulatory network (**Figure 7A**). Expanding our analysis, we identified 56 putative TFs using the hTFtarget and ChipBase databases. This led to the construction of a TF-mRNA network comprising 59 nodes and 76 edges. Notably, key regulators such as POLR2A, RUNX3, and EP300 were identified as joint regulators of biomarkers, thereby holding substantial promise (**Figure 7B**). Moving forward, we employed the CTD database to predict 61 chemicals, assembling a regulatory network comprising 79 edges. Noteworthy chemicals such as Acetaminophen, Benzo(a)pyrene, and Lipopolysaccharides were found to target all three genes, accentuating their potential relevance (**Figure 7C**). Subsequent disease prediction utilizing the DisGeNET and MalaCards databases unveiled a complex disease-gene network comprising 3 biomarkers, 58 diseases, and 77 edges. Diseases such as rheumatoid arthritis, ulcerative colitis, asthma, and Alzheimer’s disease emerged as co-occurring ailments potentially correlated with IRP and RA (**Figure 7D**). Finally, leveraging the ENCORI database, we forecasted RBPs binding to biomarkers, resulting in a regulatory network featuring ITGAX, PTPRC, 26 RBPs, and 31 edges. While the network did not predict RBP binding to HLA-DRA, several RBPs including IGF2BP1, IGF2BP3, RBM4, RBM39, and ELAVL1 exhibited potential simultaneous binding to both ITGAX and PTPRC (**Figure 7E**).

## Discussion

For the first time, we analyzed the causal relationship between RA and PAP using the two-sample bidirectional MR method. Our analysis revealed that RA significantly increased the risk of PAP (OR = 1.1284), aligning with previous findings. Julia et al. conducted a systematic review assessing the risk of developing periapical lesions in patients with autoimmune diseases and found that periapical lesions were associated with three autoimmune diseases: type 1 diabetes, RA, and inflammatory bowel disease (30). Previous studies have also demonstrated that the prevalence of AP in patients with autoimmune diseases was significantly higher than in control groups, indicating that autoimmune diseases considerably increase the risk of AP (31, 32). In a cross-sectional study involving 96 participants (48 RA patients and 48 healthy controls), 45 out of 1026 teeth in the RA group had AP, compared to 21 out of 1025 teeth in the control group. The prevalence of AP in the RA group, with at least one affected tooth, was significantly higher (47.9%) than in the control group (29.7%), with an OR of 3.087. This indicated that RA patients were more likely to develop AP compared to control patients (17). When pulpitis extends through the apical foramen, it triggers a series of inflammatory reactions, attracting inflammatory chemical mediators that cause periapical lesions and can eventually lead to periapical abscess, periapical granuloma and periapical cyst (33). Another retrospective study found that the prevalence of periapical abscesses in RA patients was 1.53%, compared to 0.51% in control patients, with an OR of 2.60. Moreover, the incidence of periapical abscesses in patients treated with Etanercept (a TNF-α inhibitor) was significantly lower than in those treated with methotrexate or salazopyridine, suggesting that TNF-α inhibitors can reduce the incidence of periapical abscesses in RA patients (34). However, our results did not confirm that PAP increases the risk of RA, and no previous studies have explored this causal relationship. It was well established that the progression of autoimmune diseases was significantly influenced by systemic inflammatory conditions (35). Periodontal disease has been reported to be associated with the risk of RA. The periodontal pathogen *Porphyromonas gingivalis* produced peptidyl arginine deaminase, which exacerbated RA pathology. Furthermore, periodontal pathogen DNA has been detected in the synovial tissue of RA patients with periodontitis, suggesting that periodontal bacteria were involved in RA (36). The similar pathogenesis of PAP and periodontitis suggested that infection control of PAP was as important as periodontitis in maintaining general health (18). However, more research was needed to understand the effect of PAP on RA fully.

Secondly, we analyzed the common DEGs and regulatory pathways associated with IRP and RA using multiple public datasets. Our analysis revealed that these two diseases share 84 DEGs, predominantly involved in immune and inflammatory pathways, including cytokine signaling, chemokine signaling, IL-17 signaling, Th17 cell differentiation, and TNF signaling pathways. Previous studies have reported similar distributions of cytokines, such as IL-1, IL-6, IL-12, IL-17, and TNF-α, in the pathological changes of RA and AP (37–41). The similar pathobiology of these diseases may explain the significant link between them. Moreover, as early as 1975, researchers identified free plasma cells producing rheumatoid factors in periapical lesions in 6% of rheumatoid patients and 4% of control patients (15). In the SKG mouse model, oral infection with *Porphyromonas gingivalis* induced Th17 cells to invade the joint cavity, and the migration of polymorphonuclear neutrophils and fibroblasts induced by IL-17 suggested a potential association between oral bacteria and RA. The inhibition of synovial cell apoptosis was considered a pathological feature of RA. A significant number of apoptotic cells were detected in SKG mice with PAP compared to similar lesions in normal mice, indicating that the phagocytic inhibitory feature of RA may also occur in oral lesions. Dysphagocytosis in RA affected infiltrating cells in PAP (18).

Using CytoHubba and machine learning methods, we identified three hub genes in IRP: HLA-DRA, ITGAX, and PTPRC. These genes were significantly overexpressed in both IRP and RA, and exhibited a strong correlation with each other. The ROC value for these genes was higher than 0.9 in IRP and higher than 0.7 in RA, indicating their high diagnostic value. HLA-DR, of which HLA-DRA is a subunit, is known to be involved in inhibiting tumor growth and plays a crucial role in human cancer (42). HLA-DRA was overexpressed in hepatocellular carcinoma, colorectal cancer, and cervical cancer, but was decreased in breast cancer. It was related to the tumor microenvironment and can predict immunotherapy reactivity (43). Liu et al. used bioinformatics methods to identify nine genes, including HLA-DRA, to create a model that can effectively distinguish RA from normal samples (44). In Sjögren’s syndrome, HLA-DRA was significantly upregulated in salivary gland epithelial cells. However, there have been few studies on HLA-DRA in PAP (45). ITGAX-positive cells have been detected in the synovial tissue of RA joints, indicating its involvement in RA inflammation. ITGAX also participated in the inflammation associated with asthma (46, 47). Liu et al. confirmed the importance of ITGAX as a diagnostic marker in IRP through bioinformatics and RT-PCR methods, and found that ITGAX was expressed more in inflamed pulp compared to normal pulp (48). PTPRC encodes protein tyrosine phosphatase, a signaling molecule that regulates various cellular processes and plays a key role in the immune system. PTPRC negatively regulated cytokine receptor signaling by inhibiting the JAK signaling pathway (49). The expression level of PTPRC was low in normal pulp tissues but high in pulpitis tissues (50, 51). Additionally, Cui et al. found that the RA risk allele PTPRC was associated with response to anti-TNF α therapy (52). PTPRC rs10919563 was a proven RA susceptibility locus, and the RA risk alleles were associated with an improved response to treatment. PTPRC has become the most replicated genetic biomarker for TNFi response (53). MR analysis revealed that RA significantly increases the risk of PAP and uncovered a correlation between RA and IRP through bioinformatics analysis. This finding suggests that the prevention and early treatment of RA could reduce the risk of PAP and provide valuable guidance for managing patients with both RA and PAP. Thus, early diagnosis and treatment of RA are critical, particularly since patients with autoimmune diseases often require lifelong immunotherapy, which can be associated with severe adverse reactions and side effects. In recent years, RA treatment has evolved, with biological disease-modifying anti-rheumatic drugs (DMARDs) such as TNF-α inhibitors, CTLA-4 inhibitors, and small molecule targeted DMARDs being recommended for patients who do not respond well to traditional DMARDs (54). HLA-DRA has emerged as a key target in immunotherapy, previously associated with an inflamed TME in tumors and various non-tumor diseases, including COVID-19, osteoarthritis, and dry eye syndrome (43, 55–57). Moreover, HLA-DRA has been identified as a prospective biomarker for clinical outcomes (58, 59), and its targeting has been widely adopted in the immunotherapy of various tumors (43, 60). Therefore, targeting HLA-DRA in RA could represent a novel therapeutic approach.

In both RA and pulpitis, immune cell infiltration is a prominent feature, particularly the recruitment of myeloid cells such as macrophages and dendritic cells. PTPRC, encoding CD45, is a protein expressed on all white blood cells and plays a critical role in immune cell signaling. Its expression confirms the extensive presence of immune cells in these diseases. CD45 influences the recognition and activation of immune cells by regulating T cell receptor and B cell receptor signaling (61). Given its central role in immune cell signaling, CD45 has emerged as a potential target for immunotherapy, with biological agents targeting CD45 being investigated to modulate immune responses (62). The protein CD11c, encoded by ITGAX, is an integrin α X chain expressed on myeloid cells (63). In RA, CD11c expression is noted in myeloid cells within the synovium, where it plays a role in the inflammatory response and antigen presentation (64, 65). The relationship between MHC-II and myeloid cells is crucial in both RA and pulp periapical diseases. MHC-II molecules, expressed on these cell types, are essential for the activation of CD4+ T cells, which are key players in anti-tumor immunity and autoimmune diseases (66). In RA, macrophages and dendritic cells within the synovium express MHC-II and are actively involved in antigen presentation (67). These findings suggest that both diseases involve increased recruitment of myeloid cells, a hallmark of Th17 signaling (68). The Th17 signaling pathway is closely associated with the initiation and progression of various inflammatory and autoimmune diseases (69). In RA patients, there is an increase in Th17 cells and the Th17/Treg ratio, along with a significant rise in CD11c+ dendritic cells, which correlates positively with the Th17/Treg ratio (70). Xu et al. found that in sepsis survivors, HLA-DRA levels gradually increased, and the Th17 levels were significantly higher compared to non-survivors (71).

In summary, this study was the first to analyze the causal relationship between RA and PAP using the MR method, finding that RA can significantly increase the risk of PAP. Additionally, we explored and identified common DEGs and regulatory pathways between RA and IRP. We identified hub genes shared between the two diseases and constructed multiple regulatory networks based on these hub genes. The genes HLA-DRA, ITGAX, and PTPRC may serve as potential biomarkers for RA and IRP, highlighting their association with both conditions. However, this study has several limitations. Firstly, the MR findings were primarily based on European populations, and further research was needed to determine their applicability to other populations. Secondly, while the MR-Egger and IVW methods suggested the possibility of PAP increasing the risk of RA, there was a lack of evidence from other methods, and large-scale retrospective or cohort studies were insufficient. Future single-center retrospective studies could explore this reverse causality in greater detail. Thirdly, although we used reasonable screening methods to explore the hub genes of RA and IRP, the difficulty in obtaining IRP pulp samples and the lack of publicly available datasets in the GEO, necessitate future collection of blood or tissue samples from patients to verify the expression and potential functions of these hub genes.

## Conclusion

This study confirmed that RA can significantly increase the risk of PAP by MR method, but the effect of PAP on RA risk was not clear. At the same time, public datasets were used to screen out the shared hub genes HLA-DRA, ITGAX and PTPRC of RA and IRP, which were up-regulated in both diseases and showed excellent diagnostic capabilities.

## Data Availability

The original contributions presented in the study are included in the article/**Supplementary Material**. Further inquiries can be directed to the corresponding author.
